# Supplementary material for: Structural dynamics of protein-protein association involved in the light-induced transition of Avena sativa LOV2 protein
Source: Nat Commun. 2024 Aug 14;15:6991. doi: 10.1038/s41467-024-51461-z (PMC11324726; doi:10.1038/s41467-024-51461-z)
Supplement: Supplementary file 1 — Supplementary Information [file 41467_2024_51461_MOESM1_ESM.pdf]

Supplementary Information

Structural dynamics of protein-protein association involved in the light-induced transition of *Avena sativa* LOV2 protein

*Changin Kim[1,2†], So Ri Yun[1,2†], Sang Jin Lee[1,2†], Seong Ok Kim[1,2†], Hyosub Lee[1,2], Jungkweon Choi[1,2], Jong Goo Kim[1,2], Tae Wu Kim[1,2], Seyoung You[1,2], Irina Kosheleva[3], Taeyoon Noh[1,2], Jonghoon Baek[1,2] and Hyotcherl Ihee[1,2]**

[1] Department of Chemistry, Korea Advanced Institute of Science and Technology (KAIST), Daejeon, 34141, Republic of Korea

[2] Center for Advanced Reaction Dynamics, Institute for Basic Science (IBS), Daejeon, 34141, Republic of Korea

[3] Center for Advanced Radiation Sources, The University of Chicago, Chicago, IL, 60637, USA

[†]These authors contributed equally to this work.

Corresponding Authors

*Hyotcherl Ihee: hyotcherl.ihee@kaist.ac.kr

Supplementary Figures

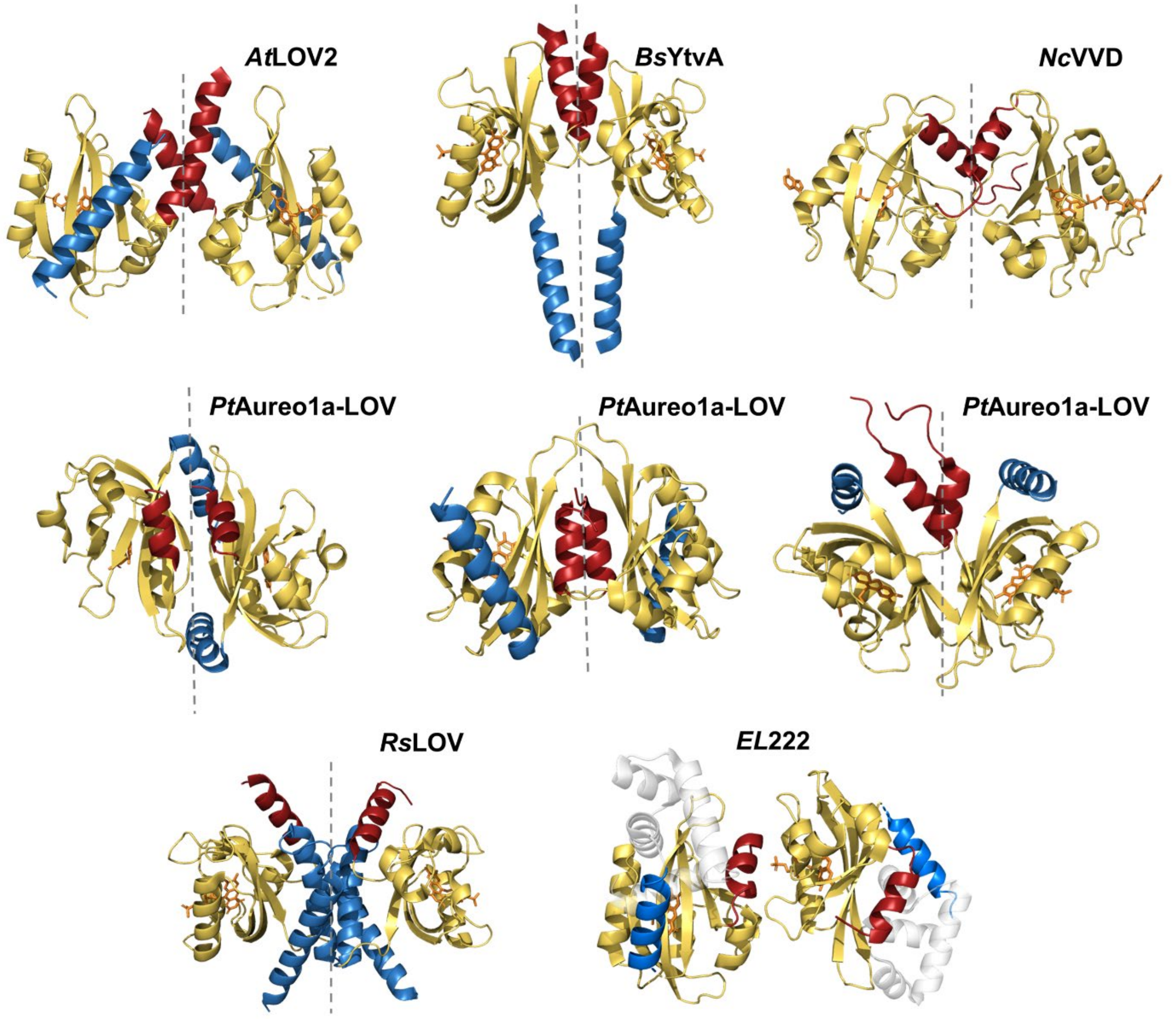

Supplementary Figure 1. Dimeric structures of LOV domains reported in the literature: *Arabidopsis thaliana* Phot1-LOV2 (*At*phot1-LOV2, PDB ID: 4HHD), *Bacillus subtilis* LOV (*Bs*YtvA, 4GCZ), *Neurospora crassa* VVD (*Nc*VVD, 3RH8), *Phaeodactylum tricornutum* Aureochrome 1a-LOV (*Pt*Aureo1a-LOV, 6T74, 5A8B, 5DKL), *Rhodobacter sphaeroides* LOV (*Rs*LOV, 4HJ3), and *Erythrobacter litoralis* 222 (*EL*222, 3P7N). The LOV domain structures are shown with the PAS core domain colored yellow, the A'α helix orange, and the Jα helix blue.

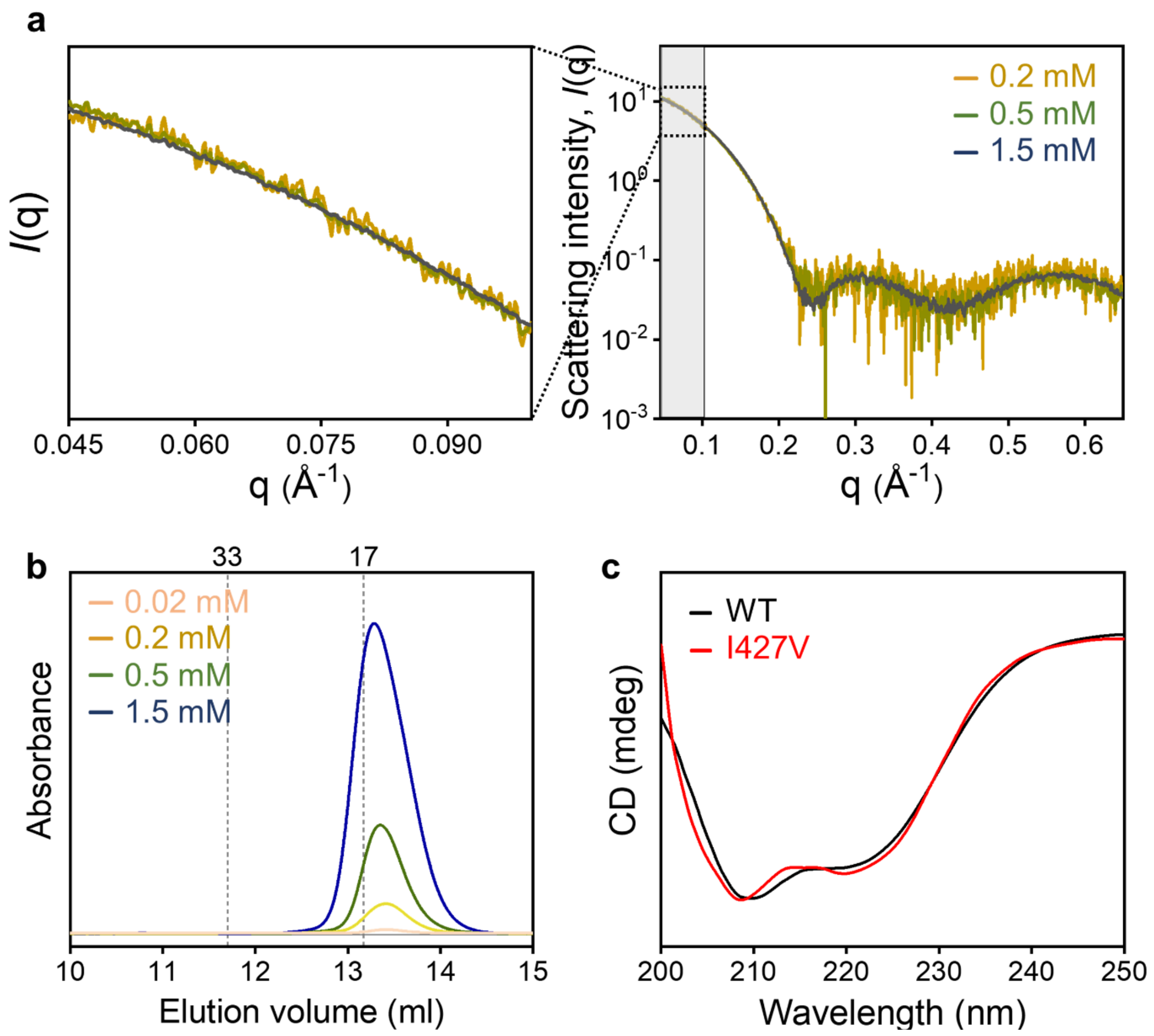

Supplementary Figure 2. Characterization of *As*LOV2. a Static SAXS curves of *As*LOV2 at concentrations of 0.2 mM (yellow), 0.5 mM (green), and 1.5 mM (blue). The left panel shows SAXS features at q < 0.1 Å^{-1}. In the small–angle regions where changes in oligomeric status can be observed, SAXS curves obtained from protein samples at these three concentrations exhibit almost identical features. **b** SEC elution profiles at the concentrations of 1.5 mM (blue), 0.5 mM (green), 0.2 mM (yellow), and 0.02 mM (light orange). **c** Circular dichroism spectra of WT (black) and I427V (red).

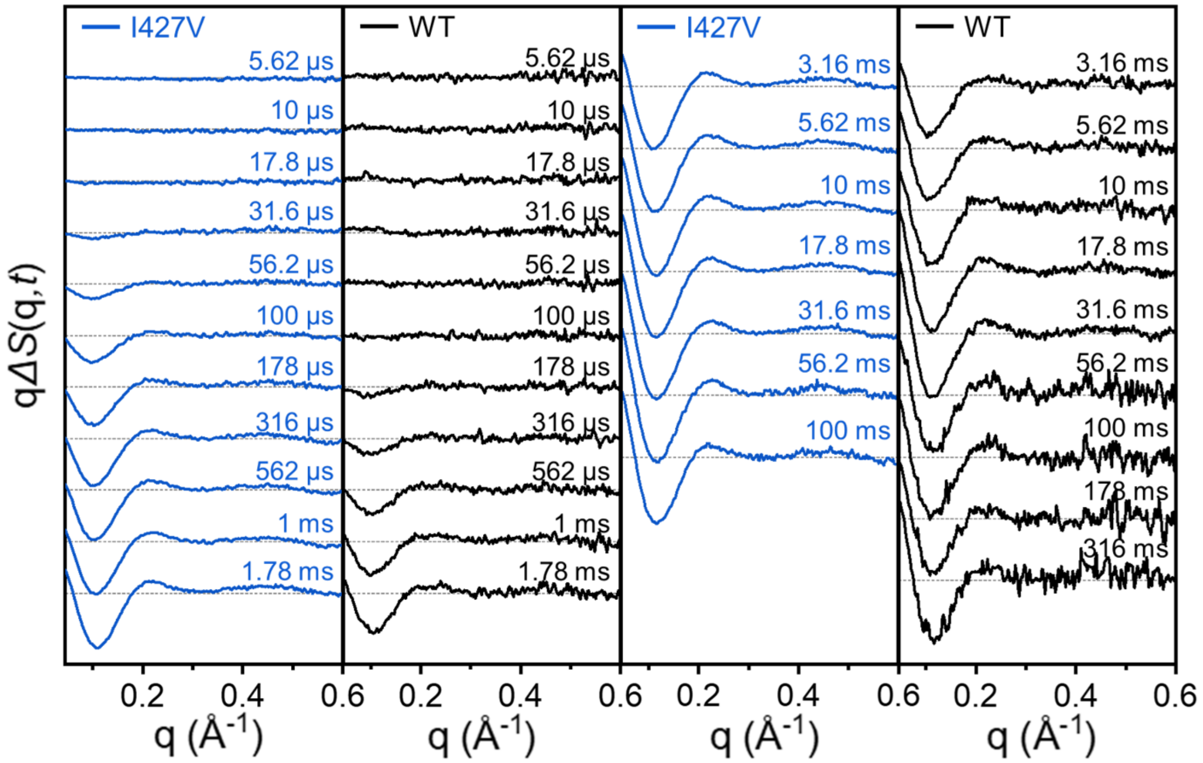

Supplementary Figure 3. TRXL data of I427V mutant and WT. The experimental difference scattering curves of the I427V mutant (blue) is compared with those of the WT (black).

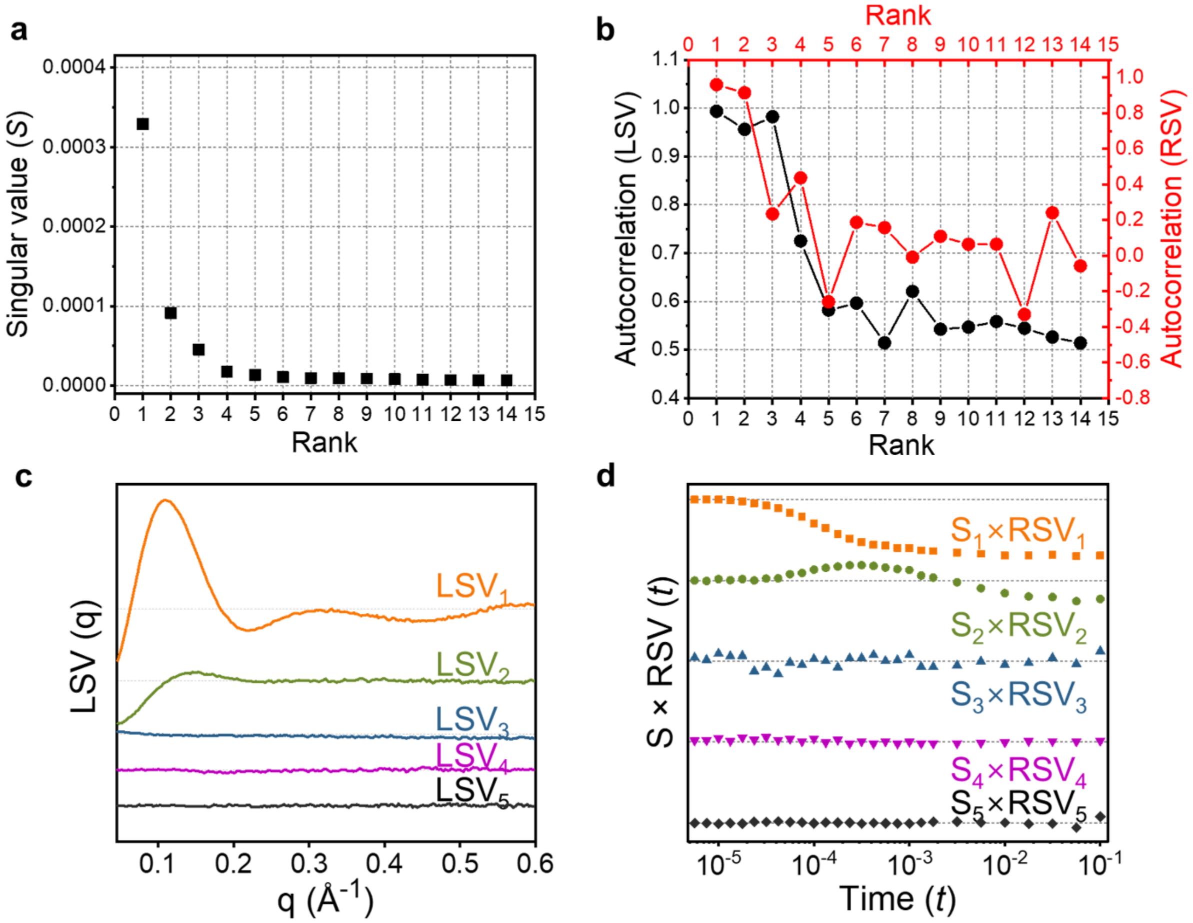

Supplementary Figure 4. SVD analysis for the TRXL data of I427V from 5.62 μs to 100 ms. a Singular values (*S*, black squares). **b** Autocorrelation values of LSVs (black circles) and RSVs (red circles). **c** First five LSVs. **d** First five RSVs. The singular values and features of singular vectors and their autocorrelation values show that the first three LSVs and RSVs are significant for the data.

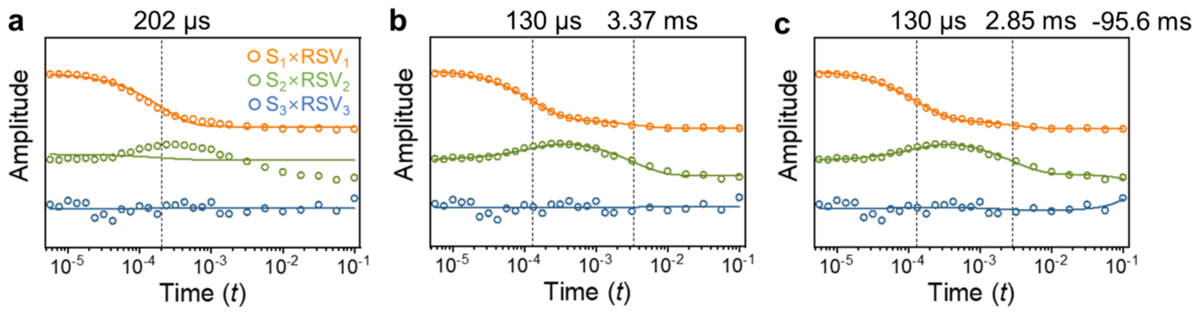

d

	τ_1	τ_2	τ_3
a	202 (± 39.9) μs		
b	130 (± 15.6) μs	3.37 (± 0.67) ms	
c	130 (± 15.4) μs	2.85 (± 0.63) ms	-95.6 (± 225) ms

Supplementary Figure 5. The fitting results of RSVs using several exponentials from the TRXL data of I427V. **a–c** Fit results of the first three RSVs multiplied by singular values using (**a**) an exponential sharing one common time constant, (**b**) exponentials sharing two common time constants, and (**c**) exponentials sharing three common time constants. The vertical dashed black lines in (**a–c**) represent the corresponding time constants determined from each fitting. **d** The time constants determined from the exponential fittings of RSVs with fitting errors.

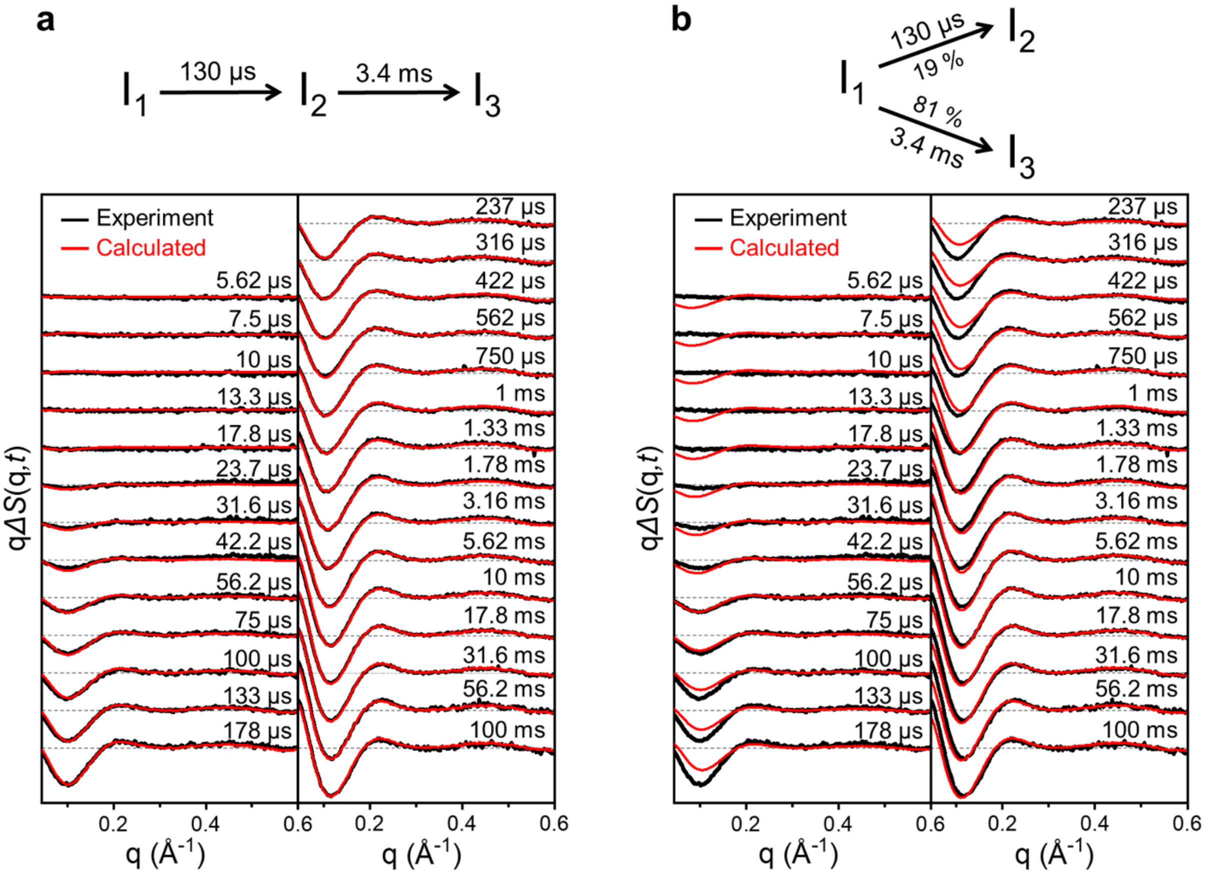

Supplementary Figure 6. Candidate kinetic models for photoreaction of I427V and comparisons of theoretical and experimental difference curves based on candidate kinetic models: a sequential kinetic model and **b** parallel kinetic model, which can be proposed based on the SVD analysis. From KCA analysis, calculated difference scattering curves were generated corresponding to the two kinetic models. Comparisons between experimental difference scattering curves (black) and theoretical difference scattering curves (red) show that the sequential kinetic model satisfactorily fits the TRXL data.

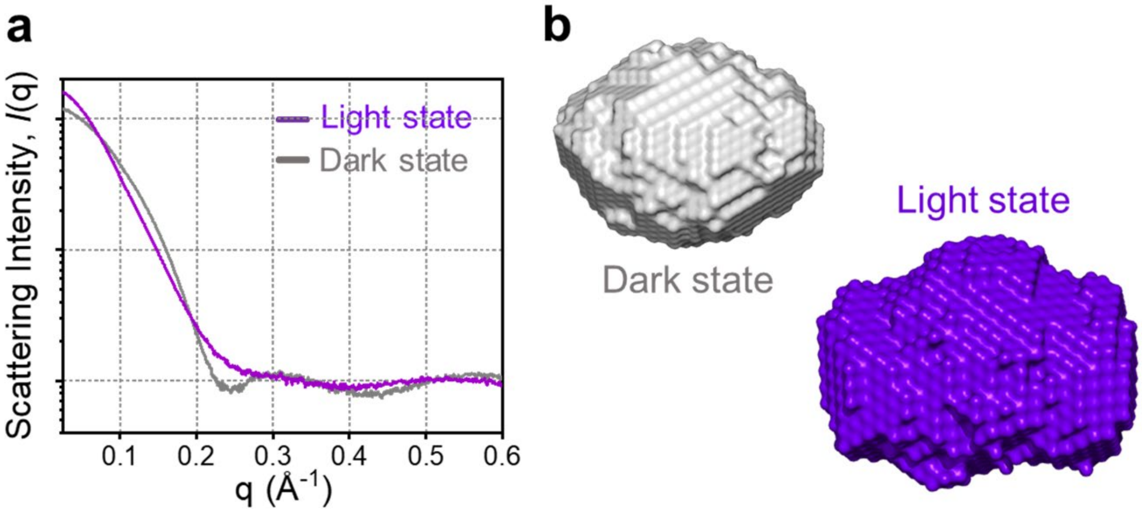

c

	Static SAXS		TRXL		
	Dark	Light	I_1	I_2	P
R_g (Å)	14.75 (± 0.02)	22.72 (± 0.03)	14.83	17.76	24.76

Supplementary Figure 7. Static SAXS data analysis. a Static SAXS curves for dark (gray) and light states (purple). **b** Low-resolution shape reconstruction models of dark state (gray) and light state (purple) from the SAXS patterns. **c** Comparison of radius of gyration (R_g) for dark, light, I_1, I_2, and P states obtained from the structural analysis on the static SAXS and TRXL data.

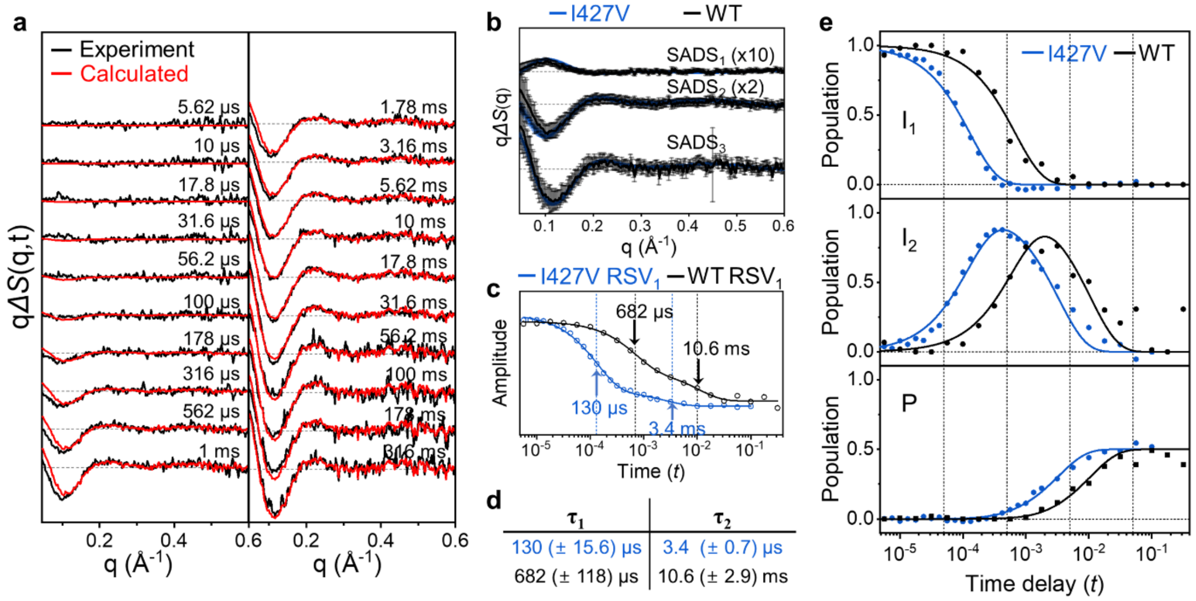

Supplementary Figure 8. Kinetic analysis on the TRXL data of WT. a Experimental scattering difference curves (black) and calculated scattering difference curves (red) of WT. **b** SADSs of WT (black). SADSs of I427V (blue) are also displayed for comparison with those of WT. The experimental errors for the SADSs of WT and I427V are represented by black and blue bars, respectively. **c** Comparison between exponential fitting results for RSV_1 of WT (black) and I427V (blue). **d** The time constants of WT (black) and I427V (blue) from the kinetic analysis on TRXL data. **e** Time-resolved population changes of I_1, I_2 and P for WT (black) and I427V (blue).

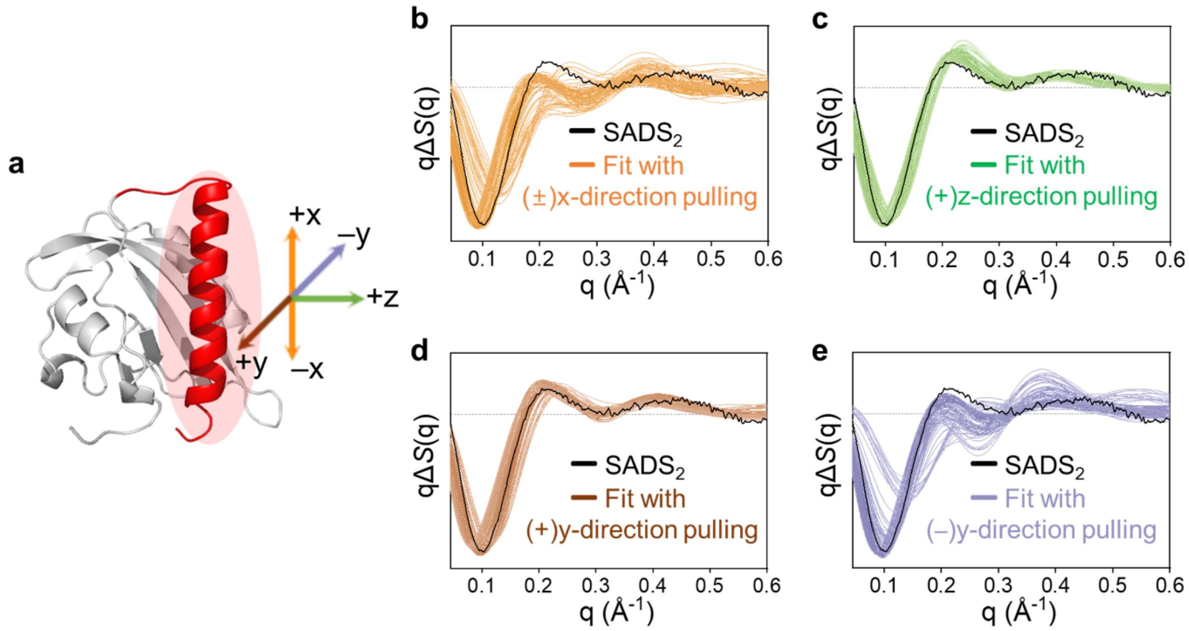

Supplementary Figure 9. Structural analysis of SADS$_2$ (corresponding to I$_2$) to describe unfolded Jα helix features. a A schematic diagram illustrating five directions (±x, ±y, and +z) for the pulling of the Jα helix. Structural analysis of SADS$_2$ was conducted using the structures obtained through NEMD simulations where the Jα helix was pulled in a specific direction (±x, ±y or +z). **b–e** Comparison of SADS$_2$ (black) with 100 well-fitted theoretical curves along the five directions: (**b**) ±x (orange), (**c**) +z (green), (**d**) +y (brown), and (**e**) –y (blue).

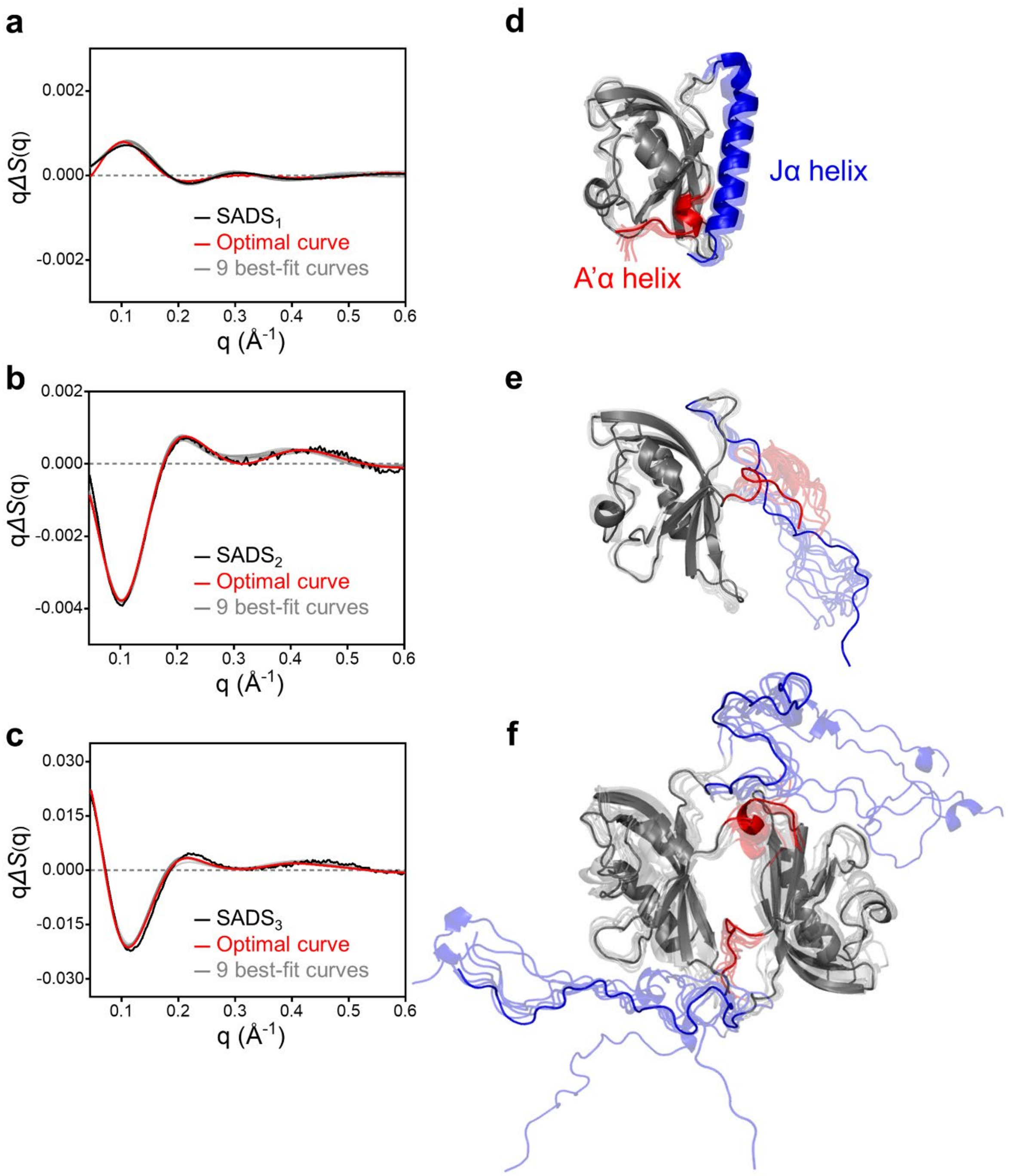

Supplementary Figure 10. Results from the MD-aided structural analysis based on the SADSs. a–c Comparison of SADS (black) with the optimal (red) and 9 best-fit (gray) curves: (**a**) I_1, (**b**) I_2 and (**c**) P. **d–f** Optimal structure and 9 best structures: (**d**) I_1, (**e**) I_2 and (**f**) P. Jα and A'α helices are depicted in blue and red, respectively, while the remaining parts are displayed in gray. In each panel of (**d**–**f**), the optimal structure is highlighted, while the rest 9 best structures are shown semi-transparently.

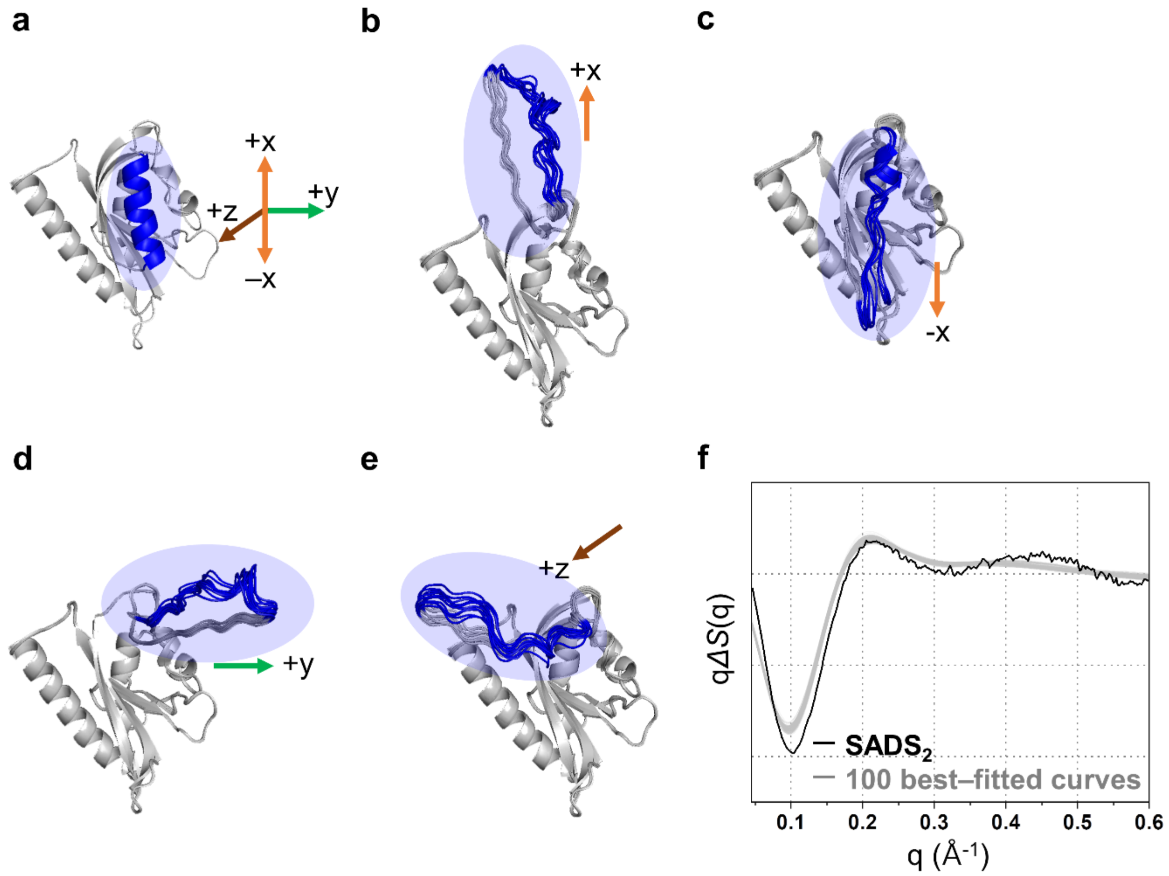

Supplementary Figure 11. Additional structural analysis of SADS$_2$ (corresponding to I$_2$) using candidate structures with unfolded Fα helix. a A schematic diagram illustrating five directions (±x, +y, and +z) for the pulling of the Fα helix. This additional structural analysis was conducted using the structures obtained through NEMD simulations where the Fα helix was pulled in a specific direction (±x, +y or +z). **b** Examples of the simulated structures with the Fα helix unfolded in the +x direction. **c** Examples of the simulated structures with the Fα helix unfolded in the -x direction. **d** Examples of the simulated structures with the Fα helix unfolded in the +y direction. **e** Examples of the simulated structures with the Fα helix unfolded in the +z direction. The structure of the Fα helix is shown in blue, while the rest of the structure is shown in gray (a–e). **f** Comparison of SADS$_2$ (black) with the 100 best-fitted theoretical curves from the candidate structures with an unfolded Fα helix, which failed to accurately describe the experimental data (gray).

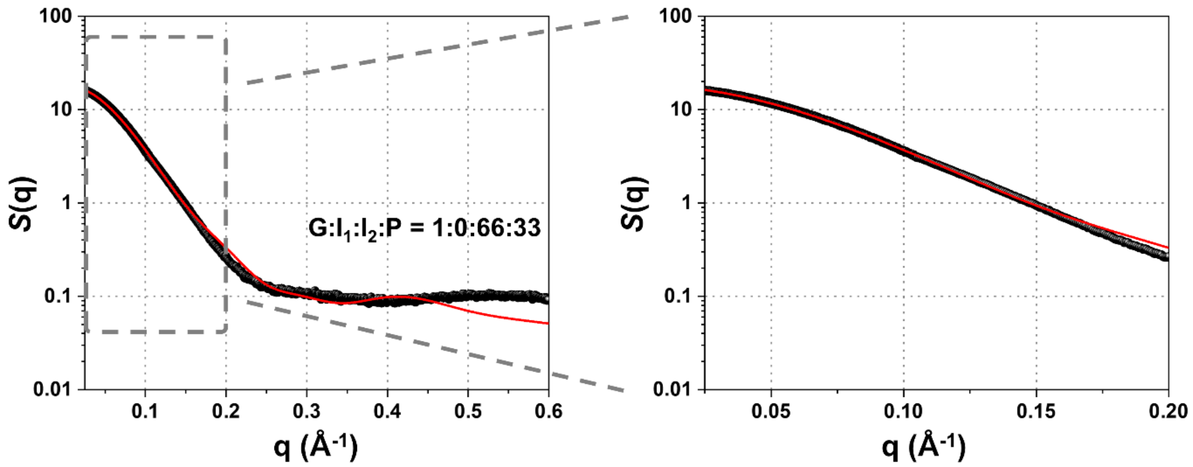

Supplementary Figure 12. Structural analysis of the static scattering curve (black) of *As*LOV2 in the light state using optimal structures obtained from analysis based on TRXL data. The theoretical curve (red) of the light state was calculated as a linear combination of theoretical curves computed using optimal structures of the ground state (G), two intermediate states (I_1 and I_2), and the photoproduct (P), with the ratio of 1:0:66:33 for G, I_1, I_2, and P.

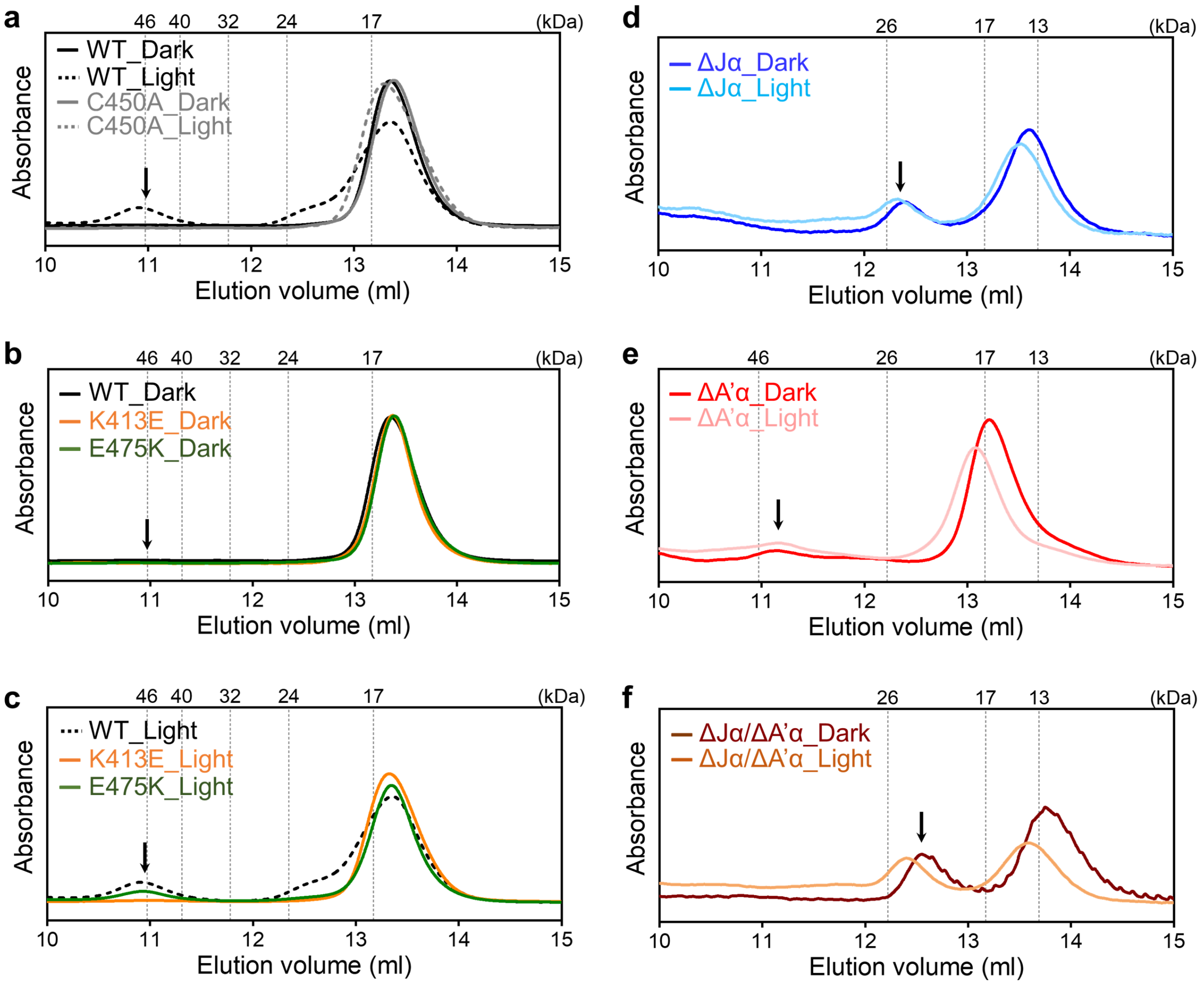

Supplementary Figure 13. SEC profiles of *As*LOV2 constructs. a Elution profiles of WT in the dark (black) and light (black dashed line) states, and C450A in the dark (gray) and light (gray dashed line) state. **b, c** Elution profiles of WT (black), K413E (orange), and E475K (green) in the (**b**) dark state and (**c**) light state. **d** Elution profile of ΔJα construct in the dark (blue) and light states (light blue). **e** Elution profiles of the ΔA'α construct in the dark (red) and light states (light red). **f** Elution profiles of ΔJα/ΔA'α construct in the dark (brown) and light states (light brown). Peaks corresponding to dimers are indicated by black arrows.

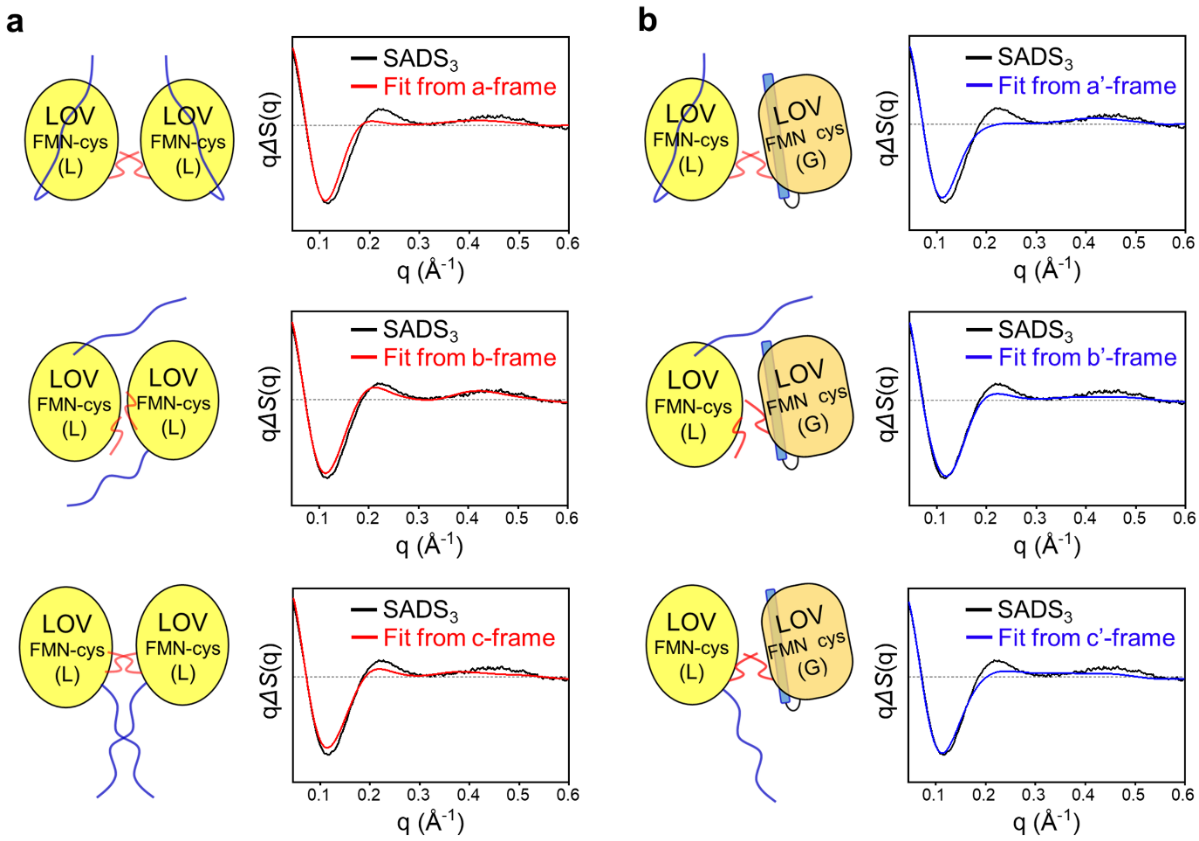

Supplementary Figure 14. Structural analysis of SADS$_3$. a, b Comparison of SADS$_3$ (black) with the best-fit theoretical curves for (**a**) three homo L/L dimer conformations with a–c frames (red), and (**b**) three hetero L/G dimer conformations with a'–c' frames (blue). Each graph is accompanied by a schematic diagram depicting the corresponding dimer conformation.

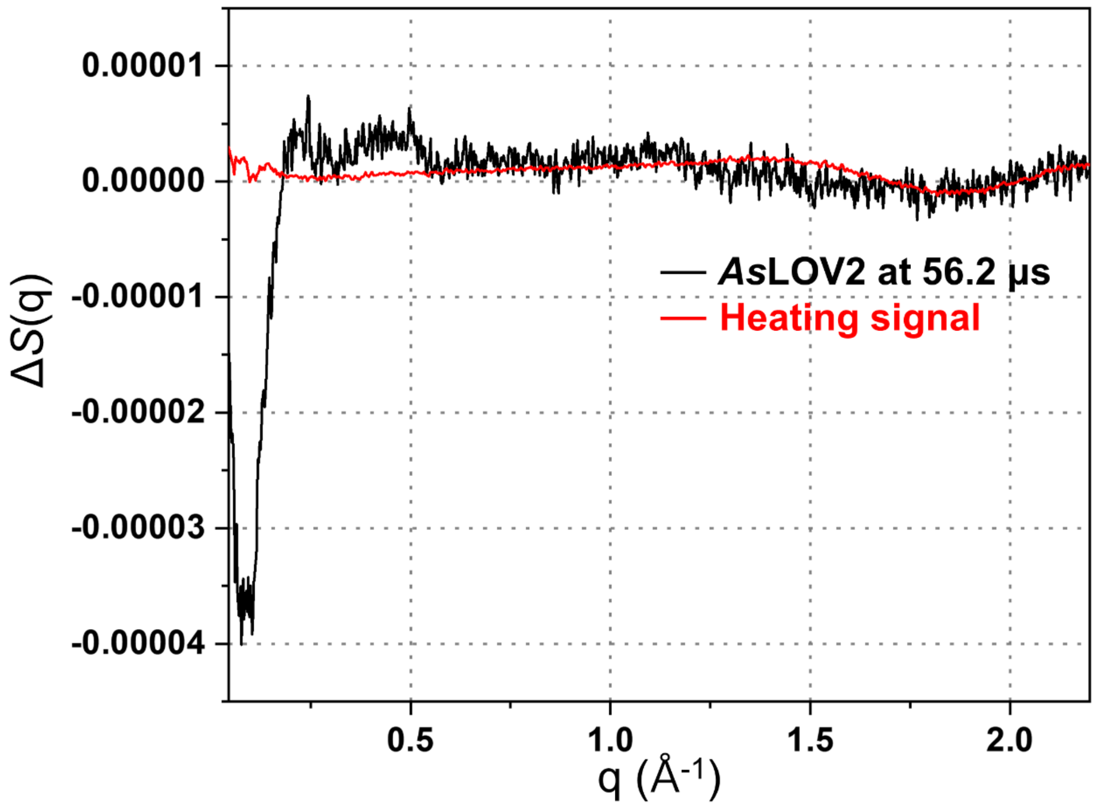

Supplementary Figure 15. Comparison of a TRXL of *As*LOV2 with a solvent heating signal. The difference scattering curve of I427V at 56.2 μs time delay (black) is compared with the solvent heating signal (red). The solvent heating signal does not show any significant features in the q-range smaller than 1.0 $Å^{-1}$.

Supplementary Tables

Supplementary Table 1. The docking simulation results for deletion constructs (ΔJα, ΔA'α, ΔJα/ΔA'α) of dark and light states, homo E/E dimer conformations (a–, b–, c–frames) and hetero E/G dimer conformations (a'–, b'–, c'–frames). Molecular docking simulations were performed to validate the possibility of dimer formation of *As*LOV2 constructs, and the plausibility of dimer formation was assessed based on the docking score (DS) and confidence score (CS).

	**Deletion constructs**						**Homo- and hetero dimer conformations**					
	ΔJα		ΔA'α		ΔJα/ΔA'α		a–frame	b–frame	c–frame	a'–frame	b'–frame	c'–frame
State	light	dark	light	dark	light	dark	light	light	light	light	light	Light
DS	−233	−217	−206	−182	−275	−275	−226	−315	−225	−199	−226	−206
CS	0.84	0.79	0.75	0.65	0.92	0.92	0.82	0.97	0.82	0.73	0.82	0.75

Supplementary Table 2. **Details for SAXS measurements and analysis.**

(*a*) Sample details		
	***As*LOV2 dark state**	***As*LOV2 light state**
Organism	*Avena sativa*	*Avena sativa*
Source (Catalogue No. or reference)	PDB: 2V1A	PDB: 2V1A
Extinction coefficient ε	ε447 nm = 13800 $M^{-1}\cdot cm^{-1}$	ε447 nm = 13800 $M^{-1}\cdot cm^{-1}$
Molecular mass *M* (Da)	16.6 kDa	16.6 kDa
Concentration	1.5 mM, 0.5 mM, 0.2 mM	1.5 mM, 0.5 mM, 0.2 mM
Solvent composition and source	20 mM Tris pH 7.0, 200 mM NaCl buffer	20 mM Tris pH 7.0, 200 mM NaCl buffer
(*b*) SAXS data collection parameters		
	***As*LOV2 dark state**	***As*LOV2 light state**
Source, instrument	4C SAXS-II beamline at Pohang Light Source (PLS-II)	4C SAXS-II beamline at Pohang Light Source (PLS-II)
Wavelength (Å)		
Beam geometry (size, sample-to-detector distance)	Size (Width, Length): 400 um, 250 um, Sample-to-detector distance: 1 m and 3 m	Size (Width, Length): 400 um, 250 um, Sample-to-detector distance: 1 m and 3 m
q-measurement range ($Å^{-11}$)	0.01375~0.69483 $Å^{-1}$	0.01375~0.69483 $Å^{-1}$
Method for monitoring radiation damage, X-ray dose	Guinier analysis	Guinier analysis
Exposure time, number of exposures	Exposure time: 10, Number of exposures: 60	Exposure time: 10, Number of exposures: 60
Flow rate	20 ul/min	20 ul/min
Sample temperature (ºC)	4 ˚C	4 ˚C
(*c*) Software employed for SAS data reduction, analysis and interpretation		
	***As*LOV2 dark state**	***As*LOV2 light state**
SAS data reduction to sample–solvent scattering, and extrapolation, merging, desmearing *etc*. as relevant	ATSAS Primus	ATSAS Primus
Basic analyses: Guinier, *P*(*r*), scattering particle volume (*e.g.* Porod volume V_P or volume of correlation V_c)	ATSAS GNOM	ATSAS GNOM
Shape/bead modelling	ATSAS DAMMIF	ATSAS DAMMIF
Molecular graphics	Pymol	Pymol
(*d*) Structural parameters		

Guinier Analysis	***As*LOV2 dark state**	***As*LOV2 light state**
$I(0)$ (cm^{-1})	12.76	17.96
R_g (Å)	17.76	22.72
q-range ($Å^{-1}$)	0.0233~0.0726	0.0269~0.05716
$P(r)$ analysis	***As*LOV2 dark state**	***As*LOV2 light state**
$I(0)$ (cm^{-1})	12.42	17.08
R_g (Å)	16.07	22.28
d_{max} (Å)	54	69
q-range ($Å^{-1}$)	0.0451~0.6948	0.0451~0.6948

Supplementary Notes

Note 1. The impact of the Fα helix unfolding on the formation of I_2.

To further confirm that the unfolding of the A'α and Jα helices is the primary structural change driving the formation of I_2, we performed an additional control analysis as follows. *As*LOV2 contains four α-helices in its core structure, in addition to the A'α and Jα helices (Fig. 1b). Notably, among these core helices, the Fα helix is relatively similar in size to the Jα helix. As a control, we further investigated the impact of the Fα helix unfolding on the formation of I_2 using the same methods applied in our structural analysis. A brief explanation of this additional structural analysis is as follows.

First, we generated candidate structures with unfolded Fα helix using NEMD simulations with the pulling algorithm of GROMACS[1] in a manner similar to those applied to the candidates with unfolded Jα helix. For the NEMD simulations, we used a representative structure from the MD trajectory of the monomer structure with a folded Jα helix as the initial structure. In the simulations, we defined two residues, Arg460 (Q3) and Asn472 (Q4), representing the N and C-terminus of the Fα helix. We then employed the external pulling force such that the Q4 position was moved away from Q3, triggering the unfolding of the Fα helix. Specifically, we applied the force to pull the Fα helix along the directions of ±x, +y, and +z (Supplementary Fig. 11a). All NEMD simulations were performed using the pulling algorithm along the hypothetical reaction coordinates until the Fα helix completely unfolded. From each MD trajectory, approximately 1,000 candidate structures for a monomer structure with an unfolded Fα helix were sampled. Subsequently, theoretical difference scattering curves were calculated using these candidate structures and compared with $SADS_2$.

As shown in Supplementary Figure 11f, the theoretical difference curves from the candidate structures with an unfolded Fα helix fail to describe the $SADS_2$, implying that the unfolding of the Fα helix does not significantly contribute to the formation of I_2. Furthermore, this finding supports that the primary structural changes driving the formation of I_2 are predominantly due to the unfolding of the A'α and Jα helices.

Note 2. Structural analysis of the static X-ray scattering curve for *As*LOV2 in the light state using the optimal structures obtained from TRXL

As mentioned in the main text, we utilized the static scattering curve of the light state to perform the reconstruction of the low-resolution structure. We did not conduct the process of refining atomic coordinates that describe the static scattering curve. Instead, as we had already found optimal conformations that well depict the difference scattering curve of the light state during the structural analysis on the TRXL data, we examined whether these optimal structures could accurately describe the scattering curve of the light state. As a result, a linear combination of the theoretical curves calculated by using the optimal structures of G, I_1, I_2, and P obtained from the structural analysis of TRXL data, with the ratio of 1(± 2.8):0(± 2.3):66(± 0.6):33 (± 0.4) for G, I_1, I_2, and P, shows good agreement with the experimental static curve in the small-angle region (0.025 $Å^{-1}$ - 0.2 $Å^{-1}$), which is sensitive to global structural properties of proteins such as the oligomer state (Supplementary Fig. 12). This result indicates that under the photo-saturation condition, while most of G converts into I_2, only a portion of I_2 forms P with the dimer conformation. This result is consistent with the observed photoconversion yield of *As*LOV2, which ranges from 0.1 to 0.3, as observed in our SEC experiment results and previous works[2,3]. Furthermore, this result indicates that under the photo-saturation condition, *As*LOV2 predominantly exists as a mixture of I_2 with the monomer conformation and P with the dimer conformation. The coexistence of these two conformations is consistent with the fact that the R_g value of 22.72 Å, estimated from the static curve of the light state, is smaller than that of 24.76 Å obtained from the dimer conformation of P.

Note 3. Validation of the dimeric interface formed between the β-scaffolds using size-exclusion chromatography and docking simulations

To further investigate the structural characteristics of *As*LOV2 dimerization, we performed size-exclusion chromatography (SEC) for four constructs in both dark and light states (Fig. 4e and Supplementary Figs, 13a, 13d–f): (i) WT, (ii) C450A mutant, (iii) a construct with the Jα helix deleted (ΔJα), (iv) a construct with the A'α helix deleted (ΔA'α), and (v) a construct with both Jα and A'α helices deleted (ΔJα/ΔA'α). For (i), the SEC profile in the dark state only displays the elution volume corresponding to a monomer conformation of the protein. In the light state, approximately 15% of the monomer fraction shifts to the elution volume corresponding to a dimer conformation. These results indicate that the light-activation of *As*LOV2, existing solely as a monomer in the dark state, is followed by dimerization of the protein, consistent with the findings from our structural analysis. On the other hand, for (ii),

only the elution volumes corresponding to a monomer conformation are observed both in the dark and light state. Considering that the C450A mutant inhibits the formation of the Cys–FMN photoadduct[4,5], the absence of the elution volume for a dimer conformation in the light state confirms that the formation of the photoadduct is necessary for the dimerization. The other constructs (iii, iv, and v) form dimers at a ratio of approximately 6-15% even in the dark state, and this ratio increases to approximately 10-20% in the light state. The fact that the dark state of the constructs lacking either Jα or A'α helices has noticeable dimer portions indicates that the Jα and A'α helices in *As*LOV2 play a critical role in hindering dimerization. Particularly, the ΔJα/ΔA'α construct exhibits the highest dimer fraction in both dark and light states, suggesting that dimerization is somewhat enhanced in the absence of these two helices.

Moreover, we conducted molecular docking simulations for the dark and light states of ΔJα, ΔA'α, and ΔJα/ΔA'α (See Supplementary Methods and Supplementary Table 1). For the docking simulations, the structure with the removed helices from the G state[6] was used as an initial frame for the dark state, and for the light state, the structure with the removed helices from the optimal structure of P was used for the light state. In the docking simulation, the plausibility of dimerization was evaluated based on docking scores (DS) and confidence scores (CS)[7] (Supplementary Table 1). A more negative value of DS and a value closer to 1 of CS indicate a higher possibility for dimerization of *As*LOV2. The light state for (ii)–(iv) has higher DS (–233, –206, –275) and CS (0.84, 0.75, 0.92) values than the dark state: the DS (–217, –182, and –275) and C.S (0.79, 0.65, 0.92). This indicates that dimerization is more promoted in the light state, where the β-scaffold is exposed due to the unfolding of Jα or A'α helices, compared to the dark state, where it is shielded by Jα or A'α. Furthermore, deleting both ΔJα and ΔA'α (ΔJα/ΔA'α) results in significantly higher DS and CS values compared to those where only a single helix was removed (ΔJα or ΔA'α variants).

The structural information about dimerization obtained from these SEC profiles and docking simulations is consistent with TRXL-derived structural information, which revealed that the dimeric interface between the β-scaffolds generated by the unfolding of the A'α and Jα helices mediates *As*LOV2 dimerization. Subsequently, we conducted docking simulations for the three homo E/E dimer (a, b and c) and three hetero E/G dimer (a', b' and c') configurations of *As*LOV2 (Fig. 4a and Supplementary Table 1). The simulations for the homo E/E dimer configurations yielded DS values of –226, –315, and –225 and CS values of 0.822, 0.965, and 0.818 for a–, b–, and c–frames, respectively. For the hetero E/G dimer configurations, the simulations yielded DS values of –199, –226, and –206, along with CS

values of 0.728, 0.821, and 0.754, for a'–, b'–, and c'–frames, respectively. The DS values obtained from the homo E/E configurations demonstrate more negative values than those derived from the hetero E/G dimer configurations. Additionally, the CS values from the homo E/E dimer configurations tend to be closer to 1 compared to those from the hetero E/G dimer configurations. Considering that a more negative DS value and a CS value closer to 1 indicate a higher possibility of dimerization for *As*LOV2, the DS and CS values support that the homo E/E dimer configurations are more favorable for the formation of dimer conformation than the hetero E/G dimer configurations. These results corroborate the structural insights from the structural analysis of the TRXL data, which suggest that in hetero E/G dimer configurations, the β-scaffold of one monomer in the G state is shielded by the A'α and Jα helices, hindering the formation of a dimeric interface with the β-scaffold of the other monomer in the excited state.

Note 4. Additional interaction influencing the formation of the dimeric interface

The SEC profile of the ΔJα/ΔA'α construct in the light state shows a relatively increased ratio of dimeric conformation compared to that in the dark state. This means that even after the helices preventing dimeric interaction between the β-scaffolds have been entirely removed in ΔJα/ΔA'α, there must be additional photoinduced interactions that facilitate the dimer formation. To explore the potential contribution of these additional interactions, we examined possible interactions among the monomers within the optimal structure of P. For this purpose, we utilized PDBePISA[8], an effective tool for identifying interactions at protein interfaces (details in Supplementary Methods), and identified potential electrostatic interactions between K413, located in the loop adjacent to the A'α helix of one monomer, and E475, located in the loop adjacent to the 3rd β-sheet (Gβ) of another monomer, that may be involved in the dimeric interface formation. To test the validity of this result, we generated two mutant types, K413E and E475K, where each residue (K413 or E475) was replaced with a residue of opposite charge and measured their SEC profiles in both light and dark states (Fig. 4e, Supplementary Figs. 13b and 13c). In the dark state, the SEC profiles of both mutant types exhibit only monomeric conformation. In contrast, in the light state, the SEC profile of K413E showed a marginal ratio of dimer conformation, and the SEC profile of E475K showed a relatively lower ratio of dimer conformation compared to WT. These results suggest that inter-

monomer electrostatic interactions between loops in *As*LOV2 may also play a crucial role in the formation of the dimeric interface.

Supplementary Methods

Elimination of the solvent heating contribution from TRXL data

Time-resolved difference scattering curves contain the contributions of structural changes in protein as well as from the heating of the solvent due to energy transfer by excited protein molecules. We reconstructed the contribution of the solvent heating response as a linear combination of the X-ray scattering change resulting from the temperature change at a constant pressure and that from the density change at a constant temperature[9]. This heating contribution was then removed from the TRXL data to extract only the contributions related to the structural changes of the target protein system (Supplementary Fig. 15). By subtracting the appropriately scaled solvent heating signal, heating-free difference scattering curves were obtained for both WT and I427V of *As*LOV2 at the positive time delays.

Static small angle X-ray solution scattering (SAXS) experiments and analysis

Static SAXS profiles under dark and light conditions were collected using monochromatic X-rays with a wavelength of 16.9 keV (Supplementary Table 2). The measurements were performed at the 4C SAXS-II beamline at Pohang Light Source (PLS-II), with sample-to-detector distances of 1 m and 3 m. During the measurements, we utilized protein samples with a concentration of 1.5 mM and circulated them using a flow cell system with a 1.0 mm-thick capillary to prevent irradiation damage. The SAXS profile of the dark state was obtained from a protein sample that had been incubated in the dark for over an hour. Subsequently, we collected the SAXS profile of the light state while continuously illuminating the protein sample with LED light at a wavelength of 455 nm (M455F3, Thorlabs, USA). In each measurement, the scattering signals were accumulated with an exposure time of 10 seconds. Additionally, we measured the SAXS profile of the buffer solution to serve as a background reference, enabling us to subtract the buffer contribution from the static scattering curve of the protein samples. The SAXS patterns for the dark and light states were processed and analyzed using the ATSAS package[10]. One-dimensional scattering curves, I(q), were generated by radial averaging of the two-dimensional scattering patterns. To isolate the curves attributed solely to the protein in the dark and light states, we subtracted the scattering curve

of the buffer from those measured in these states (Supplementary Fig. 7a). Then, by subtracting the static scattering curve of the dark state from that of the illuminated state, we obtained the static difference scattering curves, $q\Delta S_{static}$, which were used to compare with $SADS_3$.

To investigate the influence of protein concentration on the dimerization of *As*LOV2 in the G state, we conducted additional SAXS measurements on *As*LOV2 at two additional concentrations: 0.5 mM and 0.16 mM in the dark state (Supplementary Fig. 2a). The SAXS patterns obtained at the three concentrations (1.5 mM, 0.5 mM, and 0.16 mM) displayed nearly indistinguishable scattering features, indicating that *As*LOV2 does not dimerize in G state regardless of concentration.

Furthermore, to obtain structural insights into both the dark and light states, we conducted low-resolution shape reconstructions. From each SAXS profile, we calculated the pair distribution function, P(*r*), using the indirect Fourier transformation method implemented in GNOM[11]. Subsequently, these P(*r*) functions were employed to perform ab initio shape reconstructions for both the dark and light states using DAMMIF[12] (Supplementary Fig. 7b).

PDBePISA simulation

To investigate the potential interactions that play a significant role in the formation of the dimeric interface of *As*LOV2, we examined possible interactions between monomers in the optimal structure of P using PDBePISA prediction analysis. The analysis for optimal P structure was conducted using the PDBePISA web server[8] (https://www.ebi.ac.uk/msd-srv/prot_int/cgi-bin/piserver), and we obtained probable electrostatic interactions within the dimeric interface between the monomers. From the analysis, the salt bridge between K413 in one monomer and E475 in the other monomer was selected as the most possible interaction on the dimeric interface.

Size exclusion chromatography (SEC) measurements

To investigate the influence of the Jα and A'α helices on the dimerization of *As*LOV2, we measured size exclusion chromatography (SEC) profiles for deletion constructs: WT, ΔJα, ΔA'α, and ΔJα/ΔA'α, in both dark and light states (Supplementary Figs. 13a and 13d–f). In addition, to explore the influence of theoretically proposed electrostatic interactions at the *As*LOV2 dimeric interface on its dimerization, we examined SEC profiles for two mutants, K413E and E475K, in both dark and light states (Supplementary Figs. 13b and 13c). As control

samples, we also measured the SEC profile of C450A in both dark and light states, which is known not to form the Cys–FMN photoadduct in the light state (Supplementary Fig. 13a).

In the dark state, we obtained SEC profiles by flowing protein samples through the SEC column (Superdex 75 Increase 10/300 GL, Cytiva, USA) at a flow rate of 0.8 ml/min. In the light state, we measured SEC profiles for protein samples pre-incubated for one hour under illumination with 450 nm LED light (M450LP1, Thorlabs, USA). During the measurements of the SEC profiles in the light state, the protein samples were continuously irradiated with a 450 nm LED.

Docking simulation

To further investigate the influence of the A'α and Jα helices on *As*LOV2 dimerization, we conducted docking simulations for both dark and light states of ΔJα, ΔA'α, and ΔJα/ΔA'α. For light states, dimer structures of ΔJα, ΔA'α, and ΔJα/ΔA'α were generated by removing the Jα helix, A'α helix, or both Jα and A'α helices from the optimal structure of P. For dark states, we initially generated an initial dimer conformation by using the reported ground-state structure (PDB ID: 2V1A) as monomer subunits and aligning two monomers according to the dimer geometry from the optimal structure of P. Subsequently, we generated dimer structures of ΔJα, ΔA'α, and ΔJα/ΔA'α constructs by removing the Jα helix, A'α helix, or both Jα and A'α helices from the initial conformation. These docking simulations for each construct were conducted using the hdock web server[7] (http://hdock.phys.hust.edu.cn/), and we obtained the top 10 predicted dimer models with a likely dimeric interface that each construct could form. Among these predicted models, those with the highest scores were considered the most plausible conformations. The plausibility of dimer formation was assessed based on the docking score (DS) and confidence score (CS) (Supplementary Table 1).

To investigate which conformation, among the six dimer conformations (a, b, c, a', b', and c') that were used in the structural analysis of $SADS_3$, is favored for *As*LOV2 dimerization, we also conducted docking simulations for these six dimer conformations. For this purpose, we performed docking simulations using the optimal structures for each dimer conformation obtained from the structural analysis of $SADS_3$, and DSs and CSs were calculated for the dimer conformations formed in each structure.

Supplementary References

1 Van Der Spoel, D. *et al.* GROMACS: fast, flexible, and free. *J Comput Chem* **26**, 1701-1718 (2005).

2 Kawano, F., Aono, Y., Suzuki, H. & Sato, M. Fluorescence imaging-based high-throughput screening of fast- and slow-cycling LOV proteins. *PLoS One* **8**, e82693 (2013).

3 Kennis, J. T. M. *et al.* Primary reactions of the LOV2 domain of phototropin, a plant blue-light photoreceptor. *Biochemistry* **42**, 3385-3392 (2003).

4 Salomon, M., Christie, J. M., Knieb, E., Lempert, U. & Briggs, W. R. Photochemical and mutational analysis of the FMN-binding domains of the plant blue light receptor, phototropin. *Biochemistry* **39**, 9401-9410 (2000).

5 Swartz, T. E. *et al.* The photocycle of a flavin-binding domain of the blue light photoreceptor phototropin. *J. Biol. Chem.* **276**, 36493-36500 (2001).

6 Halavaty, A. S. & Moffat, K. N- and C-terminal flanking regions modulate light-induced signal transduction in the LOV2 domain of the blue light sensor phototropin 1 from *Avena sativa*. *Biochemistry* **46**, 14001-14009 (2007).

7 Yan, Y. M., Tao, H. Y., He, J. H. & Huang, S. Y. The HDOCK server for integrated protein-protein docking. *Nat. Protoc.* **15**, 1829-1852 (2020).

8 Krissinel, E. & Henrick, K. Inference of macromolecular assemblies from crystalline state. *J. Mol. Biol.* **372**, 774-797 (2007).

9 Kim, T. W. et al. Combined probes of X-ray scattering and optical spectroscopy reveal how global conformational change is temporally and spatially linked to local structural perturbation in photoactive yellow protein. *Phys. Chem. Chem. Phys.* **18**, 8911-8919 (2016).

10 Manalastas-Cantos, K. *et al.* ATSAS 3.0: expanded functionality and new tools for small-angle scattering data analysis. *J. Appl. Crystallogr.* **54**, 343-355 (2021).

11 Svergun, D. I. Determination of the regularization parameter in indirect-transform methods using perceptual criteria. *J. Appl. Cryst.* **25**, 495-503 (1992).

12 Franke, D. S., D. I. DAMMIF, a program for rapid ab-initio shape determination in small-angle scattering. *J. Appl. Crystallogr.* **42**, 342-346 (2009).
