# Supplementary material for: Structural dynamics of protein-protein association involved in the light-induced transition of Avena sativa LOV2 protein
Source: Nat Commun. 2024 Aug 14;15:6991. doi: 10.1038/s41467-024-51461-z (PMC11324726; doi:10.1038/s41467-024-51461-z)
Supplement: Supplementary file 3 — Description of Additional Supplementary Files [file 41467_2024_51461_MOESM3_ESM.pdf]

Description of Additional Supplementary Files

File Name: Supplementary Data 1
Description: Primer sequences of *As*LOV2 variants

File Name: Supplementary Data 2
Description: Initial and final structures from MD-aided structural analysis

File Name: Supplementary Data 3
Description: SEC elution profiles of AsLOV2 constructs
