# Supplementary material for: Structural dynamics of protein-protein association involved in the light-induced transition of Avena sativa LOV2 protein
Source: Nat Commun. 2024 Aug 14;15:6991. doi: 10.1038/s41467-024-51461-z (PMC11324726; doi:10.1038/s41467-024-51461-z)
Supplement: Supplementary file 7 — Reporting Summary [file 41467_2024_51461_MOESM7_ESM.pdf]

nature portfolio

Corresponding author(s): Hyotcherl Ihee

Last updated by author(s): Aug 6, 2024

Reporting Summary

Nature Portfolio wishes to improve the reproducibility of the work that we publish. This form provides structure for consistency and transparency in reporting. For further information on Nature Portfolio policies, see our Editorial Policies and the Editorial Policy Checklist.

Statistics

For all statistical analyses, confirm that the following items are present in the figure legend, table legend, main text, or Methods section.

n/a	Confirmed	
☐	☒	The exact sample size (*n*) for each experimental group/condition, given as a discrete number and unit of measurement
☒	☐	A statement on whether measurements were taken from distinct samples or whether the same sample was measured repeatedly
☒	☐	The statistical test(s) used AND whether they are one- or two-sided<br>*Only common tests should be described solely by name; describe more complex techniques in the Methods section.*
☒	☐	A description of all covariates tested
☒	☐	A description of any assumptions or corrections, such as tests of normality and adjustment for multiple comparisons
☐	☒	A full description of the statistical parameters including central tendency (e.g. means) or other basic estimates (e.g. regression coefficient) AND variation (e.g. standard deviation) or associated estimates of uncertainty (e.g. confidence intervals)
☒	☐	For null hypothesis testing, the test statistic (e.g. *F*, *t*, *r*) with confidence intervals, effect sizes, degrees of freedom and *P* value noted<br>*Give P values as exact values whenever suitable.*
☒	☐	For Bayesian analysis, information on the choice of priors and Markov chain Monte Carlo settings
☒	☐	For hierarchical and complex designs, identification of the appropriate level for tests and full reporting of outcomes
☒	☐	Estimates of effect sizes (e.g. Cohen's *d*, Pearson's *r*), indicating how they were calculated

Our web collection on statistics for biologists contains articles on many of the points above.

Software and code

Policy information about availability of computer code

Data collection	Data collection was conducted with the assistance of software from the BioCars beamline in the Advanced Photon Source (APS) and the 4C SAXS II beamline in the Pohang Accelerator Laboratory (PAL).
Data analysis	The SVD analysis and exponential fitting of TRXL data was performed using built-in plugin functions of commercially available software. Specifically, the MATLAB svd function was used for the SVD analysis and the OriginLab exponential fitting function was used for exponential fitting of RSVs. For the KCA analysis, we designed code specifically for our computational environment and dataset based on the MATLAB fmincon function. Detailed explanations of the functionality and principles of this code are provided in our manuscript, along with relevant literature citations, enabling those familiar with our computational setup to accurately replicate our analysis. Recognizing that the usability of this codes may require additional integration for general users, we are willing to offer further assistance upon reasonable request. For the structural analysis, we utilized the open source software packages (GROMACS 2019.2 and ATSAS) and the MATLAB fmincon function. Additionally, the docking simulations and finding potential interactions were conducted using the HDOCK web server, and the PDBePISA web server, respectively.

For manuscripts utilizing custom algorithms or software that are central to the research but not yet described in published literature, software must be made available to editors and reviewers. We strongly encourage code deposition in a community repository (e.g. GitHub). See the Nature Portfolio guidelines for submitting code & software for further information.

Data

Policy information about availability of data

All manuscripts must include a data availability statement. This statement should provide the following information, where applicable:

- Accession codes, unique identifiers, or web links for publicly available datasets
- A description of any restrictions on data availability
- For clinical datasets or third party data, please ensure that the statement adheres to our policy

We provided TRXL data of AsLOV2 (minimum data sets in our study) in the source data format for Figs. 2a and 2b, which were utilized for the kinetic and structural analysis presented in this study. Additionally, the SAXS profiles for the dark and light states of AsLOV2 have been deposited in the Small Angle Scattering Biological Data Bank. Furthermore, the initial and final structures for the light-induced structural changes of AsLOV2, derived from MD simulations-aided structural analysis, are provided as a separate supplemental data. The SEC data for the AsLOV2 constructs and detailed information about the primers used for the preparation of the protein samples are also provided as separate supplemental data.

Research involving human participants, their data, or biological material

Policy information about studies with human participants or human data. See also policy information about sex, gender (identity/presentation), and sexual orientation and race, ethnicity and racism.

Reporting on sex and gender	Not applicable
Reporting on race, ethnicity, or other socially relevant groupings	Not applicable
Population characteristics	Not applicable
Recruitment	Not applicable
Ethics oversight	Not applicable

Note that full information on the approval of the study protocol must also be provided in the manuscript.

Field-specific reporting

Please select the one below that is the best fit for your research. If you are not sure, read the appropriate sections before making your selection.

☒ Life sciences ☐ Behavioural & social sciences ☐ Ecological, evolutionary & environmental sciences

For a reference copy of the document with all sections, see nature.com/documents/nr-reporting-summary-flat.pdf

Life sciences study design

All studies must disclose on these points even when the disclosure is negative.

Sample size	We studied a total of seven protein constructs of the target system to elucidate its structural characteristics. These constructs were selected based on the target's kinetics and anticipated structural features.
Data exclusions	No data were excluded from the analysis.
Replication	We collected each X-ray scattering dataset more than 50 times and consistently obtained reliable data throughout the process.
Randomization	In this study, the mutant types were constructed with specific objectives, such as accelerating the photocycle of the target system or promoting/inhibiting the oligomerization of the target system. Therefore, allocation into experimental groups was not random. Each mutant was specifically designed to achieve these aims, making random allocation irrelevant to this study.
Blinding	Blinding was not necessary for this study because the mutant types were constructed with specific objectives, such as accelerating the photocycle of the target system or promoting/inhibiting the oligomerization of the target system. The design of each mutant to meet the purpose made blinding irrelevant to the data collection and analysis.

Reporting for specific materials, systems and methods

We require information from authors about some types of materials, experimental systems and methods used in many studies. Here, indicate whether each material, system or method listed is relevant to your study. If you are not sure if a list item applies to your research, read the appropriate section before selecting a response.

Materials & experimental systems

n/a	Involved in the study
☒	☐ Antibodies
☒	☐ Eukaryotic cell lines
☒	☐ Palaeontology and archaeology
☒	☐ Animals and other organisms
☒	☐ Clinical data
☒	☐ Dual use research of concern
☒	☐ Plants

Methods

n/a	Involved in the study
☒	☐ ChIP-seq
☒	☐ Flow cytometry
☒	☐ MRI-based neuroimaging

Plants

Seed stocks	No
Novel plant genotypes	No
Authentication	No
